# Nutrient Removal and Recovery: Hazenite Precipitation From Livestock Manure Wastewater

**DOI:** 10.1002/wer.70529

**Published:** 2026-08-13

**Authors:** Zehra Çoban, Ahmet Demir, Ebru Koca Akkaya, Mehmet Sinan Bilgili

**Affiliations:** ^1^ Faculty of Civil Engineering, Department of Environmental Engineering Yildiz Technical University İstanbul Türkiye

**Keywords:** ammonia stripping, geochemical modeling, Hazenite, kinetic inhibition, livestock manure wastewater, phosphorus upcycling

## Abstract

This study investigates simultaneous nutrient removal and recovery from nondigested livestock manure wastewater. To manage high suspended solids, chitosan‐mediated coagulation was utilized as a pretreatment for effective solid–liquid separation. Subsequent precipitation experiments evaluated the influence of pH, initial molar ratios, temperature, and multi‐ionic interference. Optimal conditions were identified at pH 9.5, a Mg:N:P molar ratio of 1.2:1:1, and 30°C, achieving aqueous reductions of 95% for PO_4_‐P, 91% for Mg, and 97% for NH_4_‐N. The primary scientific contribution of this study is the identification of Hazenite (KNaMg_2_(PO_4_)_2_.14H_2_O) as the recovered solid phase, rather than conventional magnesium ammonium phosphate (struvite). Structural characterizations via XRD and SEM‐EDX revealed the absence of nitrogen in the recovered precipitate. These analyses indicate that the elevated background potassium and sodium concentrations drove the crystallization of this specific alkali‐magnesium phosphate. Thermodynamic and elemental analyses indicate that the extensive depletion of aqueous NH_4_‐N was primarily driven by pH‐induced ammonia volatilization rather than solid‐phase recovery. Furthermore, ionic competition and kinetic inhibition restricted both ammonium and calcium integration into the crystal lattice. These findings demonstrate that in complex, alkali‐rich effluents, the precipitation pathway fundamentally shifts from standard struvite toward Hazenite recovery, providing an alternative for nutrient upcycling into high‐value mineral phases.

## Summary

- Chitosan coagulation serves as an effective pretreatment for raw livestock wastewater, resolving suspended solids to enable mineral recovery.
- Alkaline operations (pH 9.5) drive ammonia volatilization, redirecting the crystalline precipitation pathway from standard struvite to Hazenite.
- Hazenite recovery offers a multinutrient fertilizer alternative that effectively sequesters phosphorus and potassium from livestock effluents.
- Integrating thermodynamic modeling with elemental characterization clarifies ionic phase competition, improving the predictability of mineral recovery in complex matrices.

## Graphical abstract

This study presents an optimized precipitation process for complex livestock wastewater, achieving > 90% concurrent removal of phosphorus, magnesium, and ammoniacal nitrogen. The system successfully drives targeted Hazenite (KNaMg_2_(PO_4_)_2_.14H_2_O) crystallization, recovering essential nutrients into a high‐value, slow‐release agricultural fertilizer.

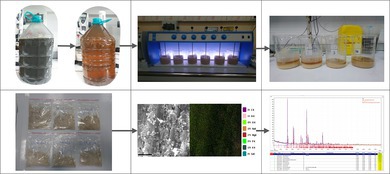

## 1 Introduction

The escalating impacts of global climate change and intensive agriculture have strained natural resources, intensifying challenges of food insecurity and freshwater contamination. Modern agriculture heavily relies on finite phosphate rock reserves, which could be depleted within the next century, positioning phosphorus scarcity as a significant threat to global food security (Li et al. 2019). Concurrently, intensive livestock farming generates massive volumes of nutrient‐dense waste, with approximately 40% of anthropogenic phosphorus losses originating from improperly managed animal effluents (Tao et al. 2016). Without adequate treatment, high nitrogen and phosphorus concentrations in these wastes can induce severe environmental consequences, including surface water eutrophication, aquatic biodiversity degradation, and ammonia emissions (Glover et al. 2023). Consequently, aligning with the 2030 Sustainable Development Goals, wastewater management has shifted toward the targeted recovery and valorization of essential nutrients (Achilleos et al. 2022).

Livestock manure wastewater is exceptionally concentrated; chemical oxygen demand (COD) can fluctuate between 10,000 and 50,000 mg/L, whereas total nitrogen (TN) and total phosphorus (TP) can reach significant levels (Vaishnav et al. 2023). An adult dairy cow produces approximately 24.5 tons of manure annually, containing 90 kg of nitrogen and 44 kg of phosphorus pentoxide (Fulhage et al. 2002). Although biological processes such as anaerobic digestion stabilize organic matter, they are often insufficient for inorganic nutrient sequestration, leaving the liquid fraction with a high nutrient load (Lorick et al. 2020; Martín‐Hernández et al. 2019; Nagarajan et al. 2023). If unmanaged, the supersaturation of these nutrients leads to mineral scaling and increased maintenance costs (Leng and Soares 2021). Extracting these macronutrients directly mitigates environmental liabilities and yields renewable secondary fertilizers, establishing a closed‐loop bio‐economy.

Among physicochemical techniques, chemical crystallization is a promising approach. Scientific literature has focused on the precipitation of standard struvite (magnesium ammonium phosphate [MAP] hexahydrate) due to its slow‐release agronomic properties (Krishnamoorthy et al. 2021). The controlled precipitation of MAP is theoretically governed by manipulating magnesium, ammonium, and phosphate ions under alkaline conditions, typically between pH 8.0 and 9.5 (Sreelakshmi et al. 2022; Tansel et al. 2018). Because raw livestock streams are inherently deficient in magnesium relative to ammonium, exogenous magnesium addition is frequently required to drive crystallization kinetics (Siciliano et al. 2020) according to the following stoichiometric expression (Rodrigues et al. 2022):

(1) $$\begin{array}{c} {\mathrm{Mg}}^{2+}+{{\mathrm{NH}}_{4}}^{+}+H_{n}{{\mathrm{PO}}_{4}}^{3-n}+{\text{6H}}_{2}O\to {\text{MgNH}}_{4}{\mathrm{PO}}_{4}.{\text{6H}}_{2}O\\ +{\mathrm{nH}}^{+}\;\left(n=0,1\;\text{or}\;2\right) \end{array}$$

However, applying crystallization to raw wastewater involves significant hurdles. The high levels of suspended solids inhibit crystal nucleation and alter reaction kinetics (Tao et al. 2016; Urbanowska et al. 2021). Physical filtration has been the standard pretreatment method (Lap et al. 2021; Le et al. 2024; Santos et al. 2024), yet, for extreme matrices like composting effluents, conventional filtration is practically unfeasible due to immediate membrane fouling and clogging. Chemical coagulation via organic biopolymers, such as chitosan, offers a robust alternative by inducing charge neutralization and polymer bridging (Ali et al. 2020). Although effective, the economic sustainability of chitosan pretreatment warrants further assessment through techno‐economic evaluations.

A critical yet underexplored challenge lies in the chemical complexity of livestock effluents. Most optimization studies utilize synthetic or municipal matrices (Kim et al. 2023; Santos et al. 2024), which may not reflect the diverse competing ions present in agricultural streams. Although the inhibitory role of calcium on MAP formation is well documented (Wang et al. 2005; Urbanowska et al. 2021; Santos et al. 2024), the influence of alkali metals, specifically potassium and sodium, remains less understood. In potassium‐rich livestock wastes (Fulhage et al. 2002), these ions may compete with ammonium during precipitation. Under the alkaline conditions required for phosphate recovery, ammonium speciation shifts toward volatile ammonia, potentially reducing its availability for MAP formation (Tansel et al. 2018; González‐Morales et al. 2021). This shift, driven by the elevated alkaline conditions (e.g., pH 9.0–12.0) requisite for crystallizing K‐rich struvite relatives from liquid wastes, promotes the formation of alternative multinutrient alkali‐magnesium phosphate phases, including K‐ and Na‐bearing compounds such as Hazenite (Magrí et al. 2025). Recent investigations further corroborate that elevated concentrations of competitive ions in such complex matrices, particularly sodium and calcium, fundamentally alter the crystallization pathway, driving the coprecipitation of these K/Na‐bearing analogues over pure single‐alkali phases (Okyere Abayie et al. 2025).

Although the optimization of operational parameters (such as pH, molar ratios, and temperature) for standard struvite precipitation has been extensively documented, the precipitation behavior in highly complex, alkali‐rich agricultural matrices remains insufficiently understood. Livestock manure wastewater frequently contains elevated background concentrations of competing alkali metals, specifically potassium and sodium, which can fundamentally alter crystallization thermodynamics. Therefore, the principal novelty of this study lies in the identification and mechanistic explanation of a distinct phase transformation occurring during nutrient recovery from chitosan‐pretreated livestock effluent. Rather than reporting standard MAP (struvite) recovery, this study elucidates how the combined effects of highly alkaline conditions, induced ammonia volatilization, and strong ion competition fundamentally disrupt conventional precipitation pathways. Consequently, the primary scientific contribution of this research is the identification and crystallographic validation of Hazenite (KNaMg_2_(PO_4_)_2_.14H_2_O) formation, providing a clearer mechanistic understanding of targeted solid‐phase nutrient recovery in complex agricultural effluents.

## 2 Materials and Methods

### 2.1 Wastewater Source and Pretreatment

The raw livestock manure wastewater was obtained from an industrial‐scale animal waste composting facility located in Yalova, Türkiye. The effluent was collected following the initial mechanical separation stage of the composting process. This liquid fraction was characterized by an extremely high nutrient load and a substantial particulate concentration (mixed liquor suspended solids [MLSSs] of 14,900 mg/L). Due to this excessive solid loading, conventional membrane or physical filtration methods were avoided to circumvent immediate fouling and clogging issues. Chemical coagulation‐flocculation was employed as a pretreatment strategy to facilitate effective solid–liquid separation. A 2% (w/v) stock solution of commercial chitosan (featuring a degree of deacetylation of 80%–85% and an average molecular weight of 3.2–2000 kDa) was prepared and dosed into the raw wastewater at a volumetric ratio of 10% (v/v), resulting in a working concentration of 2000 mg/L. The mixture was subjected to rapid agitation at 200 rpm for 5 min to induce colloidal destabilization via charge neutralization and polymer bridging.

**FIGURE 1:**
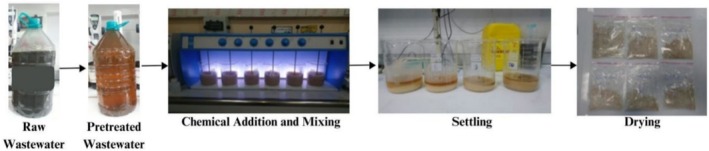
Experimental flow chart of the precipitation and recovery process.

Following the flocculation stage, the samples were centrifuged at 3400 rpm for 15 min using a Beckman Coulter Allegra X‐12 Centrifuge. The resulting clarified supernatant, hereafter referred to as the pretreated wastewater, was decanted and stored at 4°C in the dark prior to analysis and subsequent precipitation experiments. The visual transition from the raw matrix to the clarified supernatant is illustrated in Figure 1.

### 2.2 Analytical Methods and Characterization

Physico‐chemical characterization of the raw and pretreated wastewater was conducted in accordance with the Standard Methods for the Examination of Water and Wastewater (APHA 2005). The detailed analytical methodologies, inclusive of specific instruments utilized for determining pH, electrical conductivity, chemical oxygen demand (COD), and precise ionic concentrations via Inductively Coupled Plasma Optical Emission Spectroscopy (ICP‐OES, Perkin Elmer Optima and Shimadzu ICPE‐9000), are comprehensively summarized in Table 1.

**TABLE 1:** Analytical methods, instruments, and characteristic parameters of wastewater.

Parameters	Analytical methods, instruments
pH	Ohaus Starter 300 pH Meter
Conductivity	Eutech CON 510
Chloride (mg/L)	4500‐Cl [34]
Total COD (mg/L)	5220‐D [34]
TP (mg/L)	Merck Crack Set 10 UN3316
PO_4_‐P (mg/L)	Merck Phosphate Test UN3316
TKN (mg/L)	4500‐N_org_‐B [34]
NH_4_‐N (mg/L)	4500‐NH_3_‐B [34]
Mg (mg/L)	ICP‐OES Perkin Elmer Optima, Shimadzu ICPE‐9000
Ca (mg/L)	ICP‐OES Perkin Elmer Optima, Shimadzu ICPE‐9000
Na (mg/L)	ICP‐OES Perkin Elmer Optima, Shimadzu ICPE‐9000
K (mg/L)	ICP‐OES Perkin Elmer Optima, Shimadzu ICPE‐9000

### 2.3 Precipitation Experimental Design and Thermodynamic Limitations

Nutrient precipitation experiments were conducted in 1000‐mL borosilicate glass beakers using a VELP Scientifica FC6S Jar Tester. To ensure homogeneous mechanical mixing and compensate for the high initial viscosity and intense coloration of the matrix, 250 mL of pretreated wastewater was diluted with deionized water at a 1:1 ratio (total volume 500 mL). All trials were conducted in a temperature‐controlled laboratory environment with an ambient air temperature of 22°C ± 2°C under standard general room ventilation. As shown in Figure 1, all experimental trials were conducted through four sequential stages: chemical dosing, mechanical mixing, gravitational precipitation, and thermal drying.

To achieve the targeted stoichiometric molar ratios, analytical grade (MgCl_2_.6H_2_O, Merck), (KH_2_PO_4_, Merck), and ((NH_4_)HCO_3_ > 99%, Alfa Aesar) were utilized as precursors. The solution pH was adjusted using 1 N/6 N NaOH (Merck) and 1 N/6 N H_2_SO_4_. The kinetic protocol consisted of a rapid mixing phase (200 rpm, 5 min) for chemical dispersion, followed by a flocculation phase (30 rpm, 30 min) to facilitate crystal growth. Following a 60‐min settling period, the supernatant was decanted and preserved at 4°C for subsequent aqueous analysis. The recovered solid precipitate was collected and subjected to controlled thermal drying at 40°C until a constant mass was achieved. Depending on the initial moisture content, drying durations ranged between 72 and 144 h, ensuring the removal of residual moisture while minimizing thermal degradation of the hydrated phases, consistent with protocols reported in the literature (Warmadewanthi et al. 2021; Zaffar et al. 2024).

To evaluate competitive kinetics, a rigorous set of variables was tested. The pH was varied across an alkaline spectrum (7.5, 8.0, 8.5, 9.0, and 9.5). Initial molar concentration ratios of Mg:N:P were sequentially adjusted (0.8:1:1, 1.0:1:1, 1.2:1:1, 1.4:1:1, and 1.6:1:1). Temperature effects on phase solubility were investigated at 25°C, 30°C, 35°C, and 40°C (González‐Morales et al. 2021; Urbanowska et al. 2021; Sutiyono et al. 2021), utilizing a VWR 1130‐1S Circulating Waterbath. Furthermore, to examine competitive interference of foreign ions, the initial molar ratio of magnesium to calcium (Mg:Ca) was manipulated at 1:1, 2:1, and 2.5:1. Experiments were conducted in open vessels under constant stirring, with continuous monitoring of pH and temperature. Removal efficiencies were calculated based on the concentration differential between the influent (*C*_0_) and the final effluent (*C*_*e*_):

(2) $$\text{Removal Efficiency}\;\left(\%\right)=\left[\frac{C_{0}-C_{e}}{C_{0}}\right]\times 100$$

### 2.4 Crystallographic and Morphological Characterization

The phase identification of the recovered solid precipitates was conducted via X‐ray diffraction (XRD) using a Malvern PANalytical Empyrean diffractometer. The resulting diffractograms were processed using MATCH Phase Identification from Powder Diffraction software, where the figure of merit (FoM) evaluates the mathematical agreement between experimental peaks and established database references, where a value approaching 1.0 indicates a high‐confidence crystallographic match. Surface morphology and elemental mapping were visualized using a Zeiss EVO LS 10 scanning electron microscope (SEM) equipped with an energy dispersive X‐ray (EDX) spectrometer, facilitating semi‐quantitative elemental composition profiling. Supplementary microtopographical observations were performed utilizing an Olympus DP72 Polarized Optical Microscope.

### 2.5 Thermodynamic Modeling of Precipitation

Thermodynamic modeling was performed using Visual MINTEQ Ver. 4.0 (Gustafsson 2025) to evaluate the mineral precipitation potential and speciation. Consistent with the methodologies described by Cerrillo et al. (2021) and Kuşçu and Eke (2021), input parameters included the total concentrations of primary and competing ions (Mg_2_^+^, PO_4_^3−^, NH_4_^+^, K^+^, Na^+^, and Ca_2_^+^), adjusted for the 1:1 dilution ratio. Activity coefficients were calculated using the Davies equation to account for nonideal thermodynamic behavior in the high‐ionic‐strength matrix. The precipitation potential of competing mineral phases was assessed using the saturation index (SI):

(3) $$\mathrm{SI}=\log\;\left(\mathrm{IAP}\right)-\log\;\left(K_{\mathrm{sp}}\right)$$

where *IAP* is the ion activity product and *K*_*sp*_ is the thermodynamic solubility product. To accurately simulate the alkali‐magnesium pathway, the Hazenite phase (KNaMg_2_(PO_4_)_2_.14H_2_O) was manually integrated into the thermodynamic database using a solubility product (log Ks) of −24 derived from relevant aqueous phosphate studies (Yang et al. 2025). Consistent with established criteria (Kuşçu and Eke 2021), phases with an SI > 0 were considered thermodynamically oversaturated and the primary drivers of spontaneous precipitation.

## 3 Results and Discussion

### 3.1 Wastewater Characterization, Pretreatment Efficacy, and Stoichiometry

The physico‐chemical characterization of the wastewater matrix is the foundational step in determining its thermodynamic suitability for nutrient recovery. The raw livestock manure wastewater was characterized by high organic and inorganic loads, dark coloration, and a high concentration of suspended solids (Table 2). As observed from the analytical data, the majority of the total COD and TP existed in particulate or colloidal forms. Such high concentrations of suspended solids and colloidal organic matter are widely documented to severely inhibit crystallization kinetics, block crystal nucleation sites, and drastically increase the chemical demand for adjusting operational parameters (Kim et al. 2014). Consequently, direct crystallization from the raw matrix was considered thermodynamically and physically impractical.

**TABLE 2:** Physico‐chemical characterization of the raw and chitosan‐pretreated livestock manure wastewater.

Parameters	Raw wastewater	Pretreated wastewater
pH	7.3	8.1
MLSS (mg/L)	14,900	250
Conductivity	14.2	8.6
Chloride (mg/L)	—	488
Total COD (mg/L)	39,222	4872
TP (mg/L)	1400	195
PO_4_‐P (mg/L)	945	177
TKN (mg/L)	2120	1217
NH_4_‐N (mg/L)	1220	632
Mg (mg/L)	—	165
Ca (mg/L)	—	434
Na (mg/L)	—	192
K (mg/L)	—	690

The application of chitosan coagulation effectively clarified the matrix. As detailed in the methodology, the biopolymer induced charge neutralization and polymer bridging, resulting in a 98.3% reduction in MLSS (from 14,900 to 250 mg/L). This pretreatment significantly lowered the total COD to 4872 mg/L by removing particulate organic fractions, while successfully preserving the soluble nutrient pool required for subsequent precipitation. To design the chemical dosing strategy, the natural stoichiometric molar ratio of the primary ions was calculated. Utilizing the atomic weights of the target elements, initial molarities were determined as 6.79 mM for magnesium, 45.14 mM for nitrogen, and 5.71 mM for phosphorus. Normalizing these values to the limiting reagent (phosphorus) yielded a precise initial molar ratio of 1.19:7.90:1.00 (Mg:N:P). This stoichiometric baseline reveals a naturally occurring nitrogen surplus and a distinct phosphorus limitation. In studies investigating typical piggery slurry digestate, Cerrillo et al. (2021) reported comparable Mg:N:P concentrations, highlighting that although ammonium levels vary, magnesium and phosphorus concentrations in such livestock matrices often closely mirror the environment evaluated in this research.

Considering the higher molar concentration of ammonium relative to magnesium and phosphorus, the addition of external precursor ions is generally required to promote crystalline formation and obtain appreciable yields (Kwon et al. 2018). Furthermore, the initial pH of the pretreated wastewater (8.1) naturally approaches the alkaline range preferred for phosphate precipitation. However, the relatively high baseline concentrations of alkali metals, specifically potassium (690 mg/L) and sodium (192 mg/L), may introduce competitive thermodynamic effects that influence the crystallization pathways and associated phase outcomes.

### 3.2 Variables Affecting Nutrient Phase Partitioning and Removal

#### 3.2.1 The Effect of pH on Precipitation Dynamics

**FIGURE 2:**
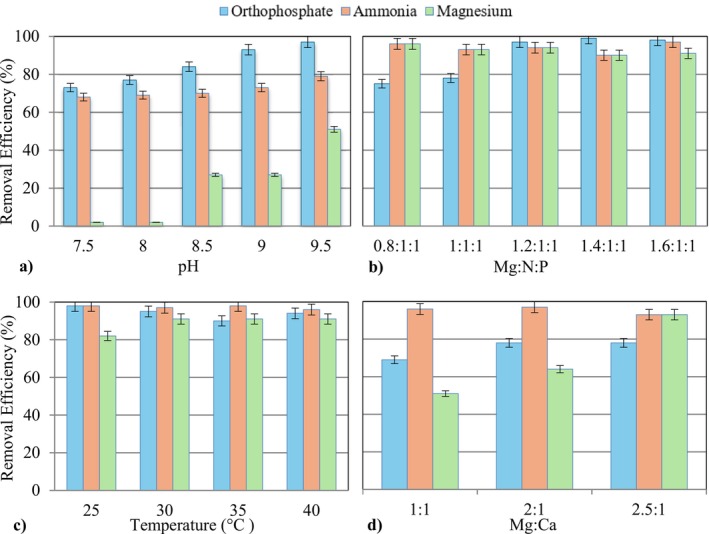
(a) Effect of initial pH on removal efficiencies of Mg, NH_4_‐N, and PO_4_‐P (Mg:N:P = 1:1:1, *T* = 25°C); (b) effect of initial Mg:N:P molar concentration ratios on removal efficiencies (pH = 9.5, *T* = 25°C); (c) effect of temperature on removal efficiencies of target ions (pH = 9.5, Mg:N:P = 1.2:1:1); and (d) effect of initial Mg:Ca molar ratios on removal efficiencies in the presence of foreign ions (pH = 9.5, Mg:N:P = 1:1:1). Optimization experiments were conducted as single experimental runs (*n* = 1). Error bars represent the analytical measurement uncertainty (approximately ± 3%) associated with the instrumental quantification methods.

Alkaline conditions, particularly pH 9.0–9.5 range, are widely documented to minimize the solubility product of magnesium phosphates and enhance precipitation kinetics (Sreelakshmi et al. 2022). To evaluate this, precipitation was executed across a pH gradient (7.5–9.5) at a baseline Mg:N:P ratio of 1:1:1 and 25°C. The results demonstrate a clear pH dependent enhancement in nutrient removal efficiencies (Figure 2a). Orthophosphate removal increased steadily from 73% to 97%, indicating a strong dependence on precipitation‐favorable speciation. Concurrently, magnesium consumption showed a marked increase from near‐negligible levels at pH 7.5 to 51% at pH 9.5, suggesting enhanced incorporation into solid phases as the solution surpassed the necessary supersaturation thresholds.

Ammoniacal nitrogen removal exhibited a moderate increase (68%–79%), with a distinct plateau at intermediate pH values followed by a further rise at pH ≥ 9.0. This trend in highly alkaline conditions raises the critical question of whether the removed nitrogen is truly incorporated into a solid phase or lost via physical mechanisms. Although several studies in the literature have attributed high ammonium depletion at elevated pH primarily to extensive incorporation into struvite crystals (Kim et al. 2014), such interpretations may not fully capture the complexity of alkaline, high‐ionic‐strength matrices. As recently demonstrated, unbalanced initial molar ratios and ion competition in complex effluents can severely restrict actual nitrogen sequestration into the lattice (Magrí et al. 2025; Okyere Abayie et al. 2025). This is consistent with observations by Urbanowska et al. (2021), who demonstrated that approximately 30% of the apparent ammonium reduction at pH 9.0 can be driven by NH_3_ desorption rather than mineralization. In the present system, the nitrogen reduction at pH 9.5 is more plausibly explained by the interplay between limited crystalline sequestration and physical mass transfer processes—specifically ammonia volatilization.

Total ammoniacal nitrogen is governed by a temperature‐dependent acid–base equilibrium (NH_4_^+^/NH_3_); at 25°C, the *pK*_*a*_ for this equilibrium is established at 9.25. When the operational pH is elevated to 9.5, surpassing this threshold, a substantial fraction (theoretically > 60%) of the available nitrogen is partitioned into the unionized, dissolved NH_3_ form. Given the open‐vessel configuration and continuous mechanical agitation, this environment facilitated the physical stripping of ammonia into the atmosphere. Therefore, the substantial nitrogen removal recorded in these trials is likely driven by volatilization rather than solid‐phase recovery. This hypothesis is supported by the subsequent absence of detectable nitrogen in the solid‐phase characterizations (Section 3.3). Consequently, while operating at elevated pH achieves efficient phosphate recovery and simultaneous ammonia removal, it appears ineffective for sequestering nitrogen into a solid, reusable fertilizer phase under these experimental conditions.

#### 3.2.2 Effect of Molar Concentration Ratios

Given that the pretreated wastewater exhibited a natural, considerably imbalanced molar stoichiometry of 1.19:7.90:1.00 (Mg:N:P), phosphate was identified as the primary limiting reagent. To drive the precipitation thermodynamics toward enhanced phosphate removal, the external addition of complementary precursor ions is generally required to rebalance the stoichiometric environment (Santos et al. 2024; Urbanowska et al. 2021). Although the theoretical ratio for standard phosphate precipitation necessitates an equimolar presence (1:1:1), increasing the magnesium‐to‐phosphate ratio raises the degree of supersaturation, thereby accelerating nucleation kinetics and facilitating the overall removal of orthophosphate and competing coions (Rahaman et al. 2008). To evaluate these dynamics, controlled Mg:N:P ratios ranging from 0.8:1:1 to 1.6:1:1 were investigated at a constant pH of 9.5 and a temperature of 25°C. As depicted in Figure 2b, a stepwise analysis of the target ions reveals distinct thermodynamic dependencies across the dosage gradient.

At lower ratios (0.8:1:1 and 1:1:1), orthophosphate removal was restricted to approximately 75% and 78%, respectively. Concurrently, magnesium consumption was high (> 93% in both scenarios). This indicates that magnesium acted as the limiting reagent; it was exhausted by competitive complexation with background ions (e.g., alkali metals), leaving insufficient magnesium to drive complete phosphate precipitation. The highest concurrent removal efficiencies were observed at moderate overdosage (1.2:1:1), achieving 97% for orthophosphate, 94% for magnesium, and 94% for ammoniacal nitrogen. This indicates that a 1.2‐M ratio compensates for competitive side reactions and provides the optimal supersaturation required for efficient phosphate capture (Ali et al. 2020; Kwon et al. 2018).

Further elevating the magnesium dosage (1.4:1:1 and 1.6:1:1) yielded a plateau in phosphate removal, signifying that the thermodynamic precipitation limit was reached. Consequently, relative magnesium efficiency slightly declined and stabilized around 90%, reflecting the accumulation of unreacted excess precursor in the aqueous phase. Previous studies evaluating anaerobic centrate or livestock effluents often report varying, nonuniform recovery rates. For instance, Kim et al. (2014) reported 99% ammonium removal but only 75% phosphorus recovery, whereas Abel‐Denee et al. (2018) achieved 82% and 67%, respectively. In this context, the uniform > 90% elimination across all target parameters at the optimal 1.2:1:1 ratio demonstrates highly effective concurrent treatment (further benchmarked in Table S1). It is worth noting that nitrogen depletion remained consistently high (> 90%) across the entire stoichiometric gradient, which is consistent with the nitrogen removal dynamics established in Section 3.2.1, suggesting that its depletion is governed by volatilization rather than being strictly dependent on magnesium‐mediated crystallization.

#### 3.2.3 Effect of Temperature on Phase Thermodynamics

Temperature exerts a complex, nonlinear influence on the solubility products of phosphate minerals. Although the solubility of typical magnesium phosphates increases between 10°C and 50°C, the crystal structure may undergo thermal degradation and phase alteration at temperatures exceeding 50°C (Uludag‐Demirer 2008; Zaffar et al. 2024). To isolate the thermal within the pretreated matrix, optimization trials were executed at 25°C, 30°C, 35°C, and 40°C, while maintaining a constant pH of 9.5 and the optimal Mg:N:P ratio of 1.2:1:1.

As depicted in Figure 2c, the influence of temperature on removal efficiencies was less pronounced than that of pH manipulation, corroborating findings in the broader literature (Tansel et al. 2018). The highest orthophosphate removal (98%) was recorded at 25°C, aligning with the thermodynamic principle that the solubility of most magnesium phosphate minerals increases with temperature (Uludag‐Demirer 2008). However, 30°C was selected as the optimal temperature to balance comprehensive multi‐ionic recovery. Whereas operating at 25°C resulted in a limitation for magnesium precipitation (82%), elevating the temperature to 30°C increased magnesium removal to 91%. Because magnesium is an essential structural cation for hazenite formation, this improvement provides a more suitable stoichiometric condition that practically offsets the minor reductions in orthophosphate (to 95%) and ammonia (to 97%) efficiencies. This trend is also consistent with findings by González‐Morales et al. (2021), who noted that although higher temperatures can accelerate reaction kinetics, they concurrently promote excessive ammonia volatilization. The slight decrease in magnesium removal efficiency as temperatures approached 40°C further suggests a minor increase in the solubility of the precipitated alkali‐magnesium phosphate phases. Consequently, maintaining the reaction at 30°C provides an optimal thermodynamic balance, facilitating solid‐phase precipitation while managing nitrogen through selective volatilization, without requiring the excessive heating energies demanded by higher temperatures. To evaluate this partitioning, the temperature‐dependent dissociation constant (*pK*_*a*_) was modeled as follows (Alhelal et al. 2022):

(4) $$pK_{a}=0.09018+\frac{2729.92}{T}$$

At the optimal operational temperature of 30°C (*T* = 303.15 K), the *pK*_*a*_ value shifts downward to 9.10. Utilizing this constant, the theoretical fraction of free ammonia (FA) within the total ammoniacal nitrogen (TAN) pool at pH 9.5 can be determined via the modified Henderson‐Hasselbalch equation:

(5) $$FA\left(\%\right)=\frac{1}{1+10^{\left(pK_{a}-\mathrm{pH}\right)}}\times 100$$

Substituting the experimental parameters (pH = 9.50, pKa = 9.10) reveals that, thermodynamically, 71.5% of the total nitrogen is partitioned into the unionized, volatile NH_3_(aq) state. During the continuous mechanical agitation in the open‐reactor setup, this substantial fraction of dissolved ammonia is physically stripped into the atmosphere. In accordance with Le Chatelier's principle, the ongoing volatilization of NH_3_(gas) continuously drives the aqueous equilibrium forward to replace the lost fraction, ultimately accounting for the observed cumulative 97% depletion of nitrogen from the liquid phase. Consequently, although lacking direct off‐gas measurements to close the mass balance, this physicochemical calculation supports the interpretation that the pronounced nitrogen loss is primarily a product of thermodynamic stripping rather than crystalline solid‐phase recovery.

#### 3.2.4 Effect of Ionic Competition on Phase Partitioning

Livestock manure wastewater is a highly heterogeneous matrix containing various ions that can interfere with targeted precipitation kinetics, influencing the reaction rate, product purity, and crystallographic identity of the precipitate (le Corre et al. 2005). In concentrated organic waste streams, calcium (Ca_2_^+^) is frequently identified as a major competitor. In calcium‐rich environments, Ca_2_^+^ tends to react with available phosphate species to form amorphous calcium carbonates and calcium phosphates, such as hydroxyapatite, rather than magnesium‐centric minerals (Santos et al. 2024). Wang et al. (2005) demonstrated that an initial Mg:Ca molar ratio of 2:0.5 yields a precipitate purity of 85%, whereas narrowing the ratio to 2:1 substantially decreases purity to 61%.

To assess this interference, the initial Mg:Ca ratio of the pretreated wastewater, naturally situated at approximately 0.63:1, was manipulated across molar ratios of 1:1, 2:1, and 2.5:1 at pH 9.5. As depicted in Figure 2d, widening the Mg:Ca ratio from 1:1 to 2.5:1 enhanced removal efficiencies. Specifically, phosphate removal increased from 69% to 78%, whereas magnesium consumption scaled from 51% to 93%. By maintaining a magnesium concentration significantly higher than calcium, the thermodynamic competition is mitigated, favoring precipitation pathways that remain magnesium‐centric (Urbanowska et al. 2021). This is consistent with the optimized Mg:N:P dosing (1.2:1:1) established in Section 3.2.2, which corresponds to an effective Mg:Ca ratio of approximately 3:1. However, a major source of ionic interference in this specific wastewater matrix appears to originate not from alkaline earth metals like calcium but rather from the substantial presence of alkali metals—specifically potassium (K^+^) and sodium (Na^+^). As established by Kabdaşli et al. (2006), the ionic strength generated by elevated alkali loads strongly influences both the induction time and the terminal morphology of the resulting crystals.

The depletion of ammonium via volatilization at pH 9.5 creates a unique environment where these abundant alkali ions encounter reduced competition for magnesium and phosphate binding sites. This specific ionic landscape suggests a potential crystallographic phase shift, pivoting the reaction away from standard MAP and toward the formation of alkali‐magnesium phosphate minerals. The exclusion of Ca^2+^ from such a potential alkali‐substituted framework can be explained by the charge‐neutrality requirements and specific lattice parameters, which preferentially stabilize monovalent alkali metals. Although the ionic radius of Ca^2+^ (1.00 Å) is strikingly similar to that of Na^+^ (1.02 Å), the framework's preference for monovalent building units effectively hinders the incorporation of divalent calcium. As suggested by Taddei (2026), this mechanism prevents the formation of amorphous calcium phosphate phases, potentially favoring a more complex, multimetal phosphate structure. The definitive crystallographic identity of this recovered phase is further elucidated through the structural analysis in Section 3.3.

### 3.3 Crystallographic and Elemental Characterization of the Solid Phase

To evaluate the identity, purity, and morphological topography of the precipitate generated under the optimal conditions (pH 9.5, Mg:N:P = 1.2:1:1, *T* = 30°C), the solid phase was subjected to structural characterization. Standard struvite is an orthorhombic crystal with a stoichiometric composition. If MAP were successfully formed, semiquantitative elemental profiling would detect distinct mass fractions of nitrogen, magnesium, and phosphorus. However, the SEM‐EDX analysis yielded a different elemental signature.

**FIGURE 3:**
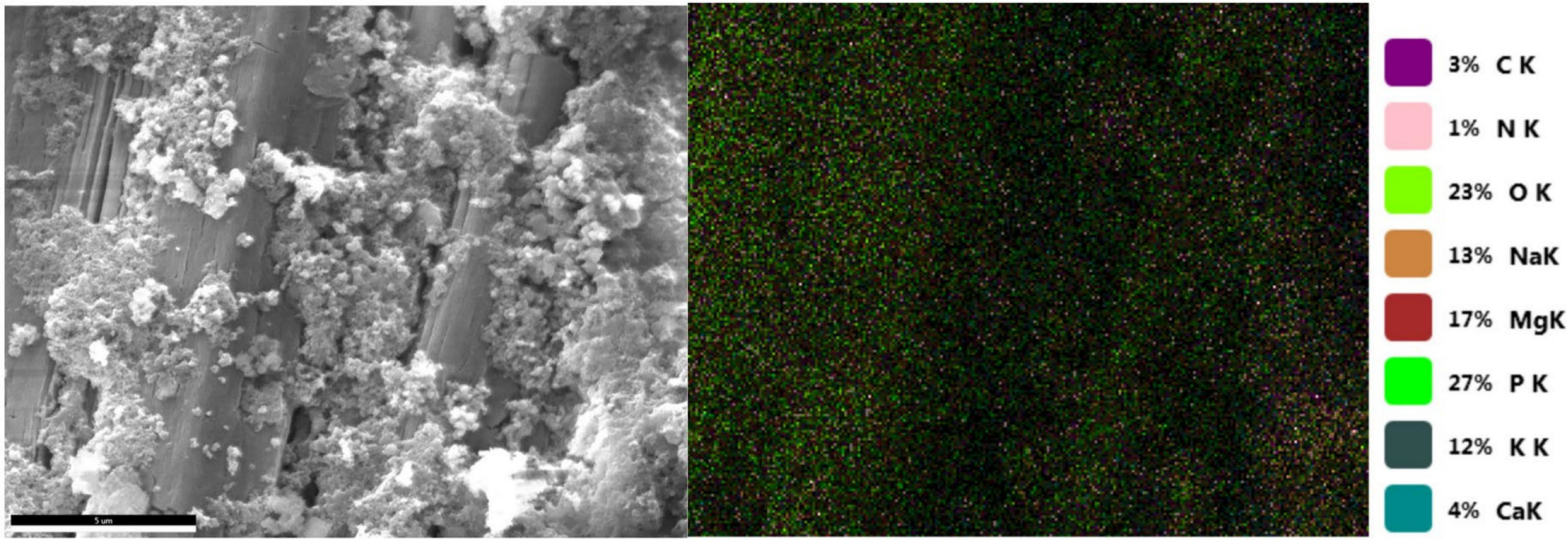
Representative EDS elemental overlay maps of the solid precipitate synthesized, highlighting the distribution of key elements.

As detailed in Table 3, the analysis revealed notable mass fractions of oxygen (37.40%), phosphorus (21.02%), potassium (12.40%), magnesium (11.93%), and sodium (10.42%). Crucially, the elemental profile demonstrates an absence of nitrogen (0.00 At%). Because standard MAP necessitates an equimolar theoretical ratio of Mg:N:P (1:1:1) within its lattice, this observation supports the thermodynamic predictions in Section 3.2.3, indicating that elevated pH and temperature drove ammonia volatilization. It should be analytically acknowledged that energy‐dispersive X‐ray spectroscopy (SEM‐EDX) possesses inherent semiquantitative limitations regarding the precise detection of low‐Z (light) elements such as nitrogen, primarily due to low X‐ray fluorescence yields and high absorption by the sample matrix. Therefore, the EDX elemental profile alone is insufficient to entirely rule out standard struvite formation. However, when this nitrogen absence is coupled with the crystallographic XRD data, which lacks the characteristic diffraction peaks of standard MAP, the exclusion of conventional struvite from the recovered solid phase becomes supported. Instead, the atomic percentages of phosphorus (14.48%), magnesium (10.47%), sodium (9.67%), and potassium (6.77%) suggest a shift toward alkali‐substituted phases. Although these proportions exhibit minor deviations from the theoretical stoichiometry of Hazenite, a common occurrence in heterogeneous wastewater due to surface adsorption or amorphous coprecipitates, they indicate the crystallographic displacement of NH_4_^+^ by K^+^ and Na^+^. Furthermore, the limited incorporation of calcium (only 1.47 At%) provides structural evidence of kinetic inhibition discussed in Section 3.2.4. Hazenite crystallizes in the orthorhombic system with the space group *Pmnb* (Yang et al. 2011). In such complex phosphate frameworks, the integration of constituent ions depends on the size of the lattice cavities. Because the effective ionic radius of Ca^2+^ (1.00 Å) is larger than that of the specific Mg^2+^ (0.72 Å) coordination sites, substituting calcium introduces steric strain. This size mismatch restricts its extensive coprecipitation within the hazenite structure, despite its presence in the matrix. As illustrated in the EDS overlay map (Figure 3), the near‐complete absence of nitrogen (1%) and the substantial spatial distribution of sodium (13%) and potassium (12%) across the scanned surface align with the formation of a complex alkali‐magnesium phosphate lattice. Although single‐alkali phases such as pure K‐struvite (MgKPO_4_.6H_2_O) can theoretically form in nitrogen‐depleted matrices, they cannot crystallographically account for this high degree of concurrent sodium incorporation. Instead, the simultaneous abundance of both monovalent cations (K^+^ and Na^+^) in the matrix may favor the stabilization of a dual‐alkali framework.

**TABLE 3:** Semiquantitative SEM‐EDX elemental composition profiling of the recovered solid precipitate.

Element	Weight %	Atomic %	Net int.	Error %	*K* ratio	*Z*	*A*
C K	4.06	7.22	8.27	15.42	0.008129915	1.1269	0.1775
N K	0.00	0.00	0.00	99.99	4.367057E‐06	1.0987	0.1700
O K	37.40	49.90	192.23	8.64	0.1274761	1.0741	0.3173
NaK	10.42	9.67	77.37	6.94	0.05772406	0.9711	0.5679
MgK	11.93	10.47	119.53	5.88	0.07591262	0.9864	0.6419
P K	21.02	14.48	196.95	3.39	0.1699852	0.9290	0.8648
K K	12.40	6.77	78.43	3.52	0.1059704	0.8928	0.9473
CaK	2.76	1.47	14.21	10.48	0.0236724	0.9080	0.9367

**FIGURE 4:**
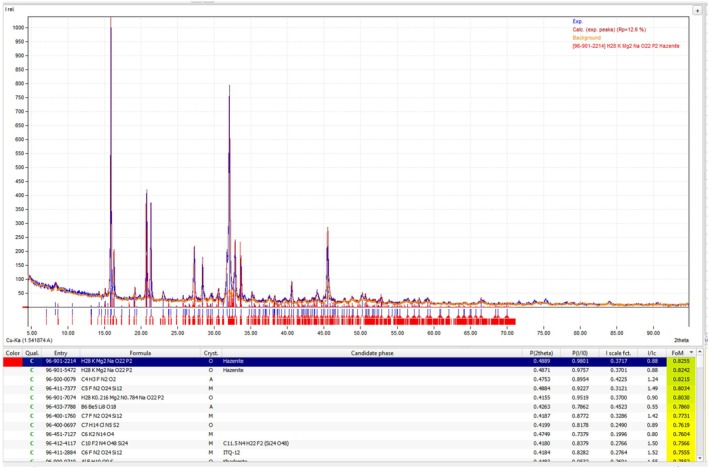
MATCH software phase identification revealing the synthesized precipitate as Hazenite.

To corroborate the EDX findings and identify the exact mineralogical phase, XRD was performed. When the experimental diffractogram was processed through the MATCH phase identification software, the peaks did not align with standard MAP. Instead, as depicted in Figure 4, the algorithm returned an optimal match with a highly hydrated, complex alkali magnesium phosphate mineral known as Hazenite (KNaMg_2_(PO_4_)_2_.14H_2_O) (Yang et al. 2011; Hazenite Mineral Data 2024). The obtained FoM score of 0.82 demonstrates a strong crystallographic correlation with Hazenite, which typically forms in nitrogen‐starved matrices where alkali metals effectively outcompete ammonium (Uysal and Yıldızbaş 2024).

**FIGURE 5:**
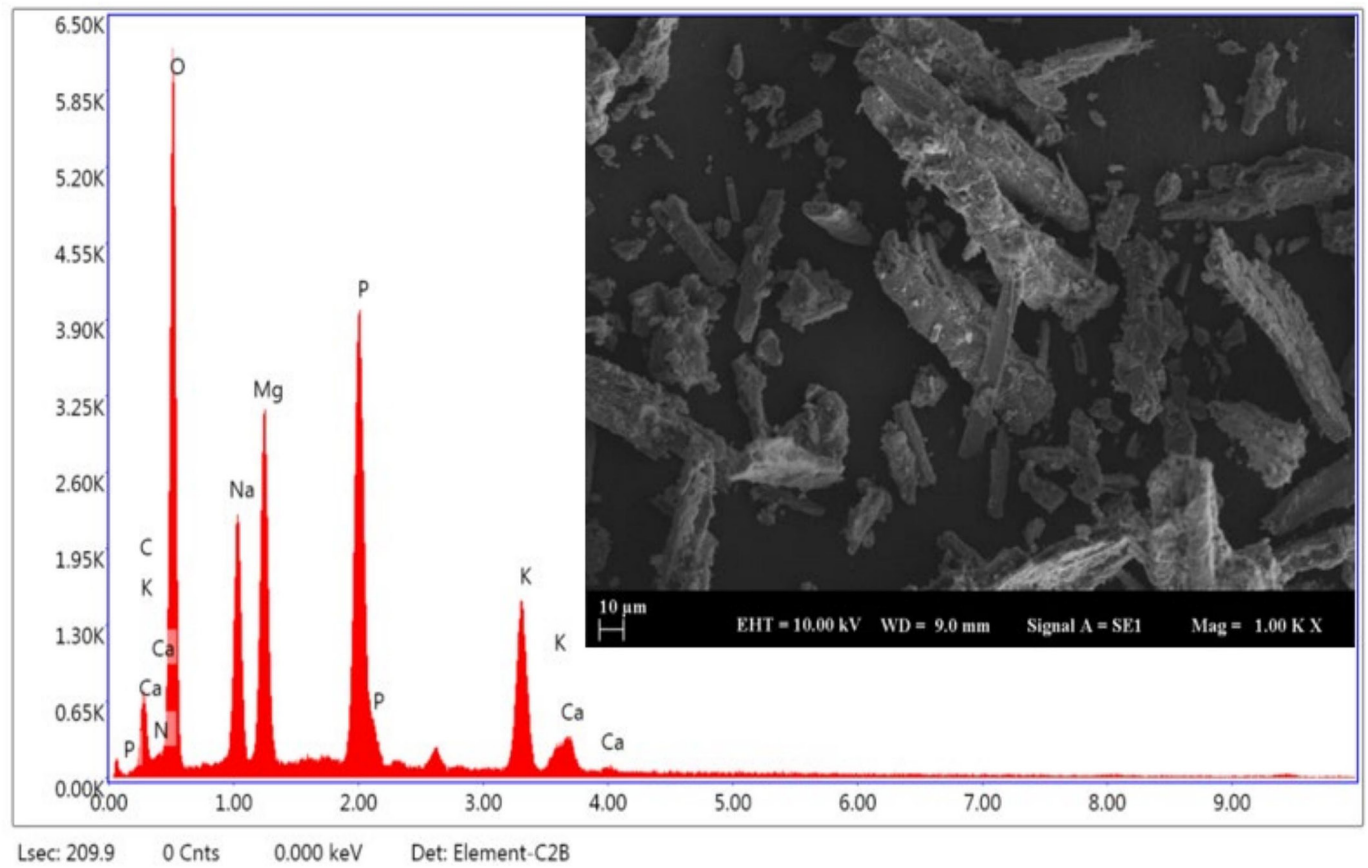
SEM images and corresponding EDX spectra of the synthesized Hazenite precipitate at optimum conditions (magnified 1.00 KX).

Further morphological validation was achieved via SEM. Standard MAP crystals classically present as distinct, well‐defined orthorhombic or coffin‐like structures (Taddeo et al. 2018; Mavhungu et al. 2020; Urbanowska et al. 2021). Conversely, the SEM micrographs obtained in this study (Figure 5), alongside polarized optical microscopy (Figure S1), reveal a morphology dominated by distorted, rod‐like rectangular prisms that display structural fragmentation and crumbling. This irregular topography is characteristic of alkali‐substituted phosphates precipitated in complex, interference‐heavy organic matrices. The lack of elemental nitrogen, the 0.82 FoM XRD match with Hazenite, and the fragmented prismatic morphology collectively provide empirical evidence that the optimized recovery process yielded a K/Na‐bearing alkali magnesium phosphate rather than standard struvite.

### 3.4 Thermodynamic Prediction of the Precipitation Pathway

To simulate the thermodynamic propensity of the complex alkali‐magnesium phosphate phase, Hazenite (KNaMg_2_(PO_4_)_2_.14H_2_O) was manually integrated into the Visual MINTEQ database. The standard solubility product of hazenite (*logK*_*s*_ = −24) was adopted from recent studies investigating aqueous phosphate systems (Yang et al. 2025). The simulation results under optimized conditions indicated a thermodynamic driving force favoring alkali‐substituted phases. Notably, the SI for Hazenite was determined to be 2.097 (Table S2), which exceeded the theoretical precipitation potential of standard struvite (SI = 1.375).

Although simple binary magnesium phosphates (e.g., Mg_3_(PO_4_)_2_(s), SI = 2.828) also exhibited high theoretical supersaturation, the structural integration of K^+^ and Na^+^ confirmed by earlier EDX analysis that the actual crystallization pathway favored the complex alkali‐magnesium lattice of Hazenite. The identification of Hazenite necessitates the formal exclusion of alternative competitive phases. First, an entirely amorphous or mixed Mg‐phosphate precipitation pathway can be confidently ruled out; the experimental XRD diffractogram exhibited multiple distinct and sharp peaks (e.g., at 2 theta = 15.6°, 20.4°, and 32.0°), indicative of a ordered crystalline structure rather than the characteristic broad halos of amorphous solids. Second, although single‐alkali substituted phases such as K‐struvite or Na‐struvite are thermodynamically possible in nitrogen‐depleted matrices, they were not the primary product. The initial pretreated wastewater contained substantial concentrations of both potassium and sodium. This simultaneous abundance of both monovalent cations thermodynamically favors the crystallization of Hazenite over pure K‐struvite, as Hazenite's complex orthorhombic spatial framework accommodates the synergistic coprecipitation of both alkali metals into a single, highly hydrated lattice. This is consistent with previous observations regarding alkali ion competition in high‐strength waste streams (Cerrillo et al. 2021; Warmadewanthi et al. 2021). Furthermore, although the thermodynamic model predicted pronounced supersaturation for a broad spectrum of calcium phosphate phases, ranging from amorphous precursors (Ca_3_(PO_4_)_2_ (am), SI > 5.3) to highly stable hydroxyapatite (SI = 21.917), the experimental data confirmed limited calcium incorporation.

This discrepancy between thermodynamic equilibrium predictions and experimental observations can be attributed to kinetic inhibition mechanisms. According to Ostwald's step rule, the precipitation of stable calcium phosphates requires the intermediate formation and structural rearrangement of amorphous phases. In complex wastewater matrices, this kinetic pathway is frequently inhibited by elevated Mg^2+^ concentrations, which can block the crystal growth sites of calcium phosphates, and residual dissolved organics that coat the nucleation nuclei (Kwon et al. 2018; Gao et al. 2024). Consequently, the kinetic suppression of calcium phases, combined with the depletion of the NH_4_^+^ precursor via volatilization at pH 9.5 (Kuşçu and Eke 2021), appears to have facilitated the selective precipitation of Hazenite. This thermodynamic landscape is in strong agreement with the selective recovery of phosphorus into a K/Na‐bearing mineral phase, as evidenced by the solid‐phase characterization results.

### 3.5 Implications for Sustainable Waste Valorization and Methodological Limitations

Recovering Hazenite instead of standard MAP offers a distinct alternative for the valorization of complex livestock effluents. The recovered mineral, containing 12.40% K, 21.02% P, and 11.93% Mg by weight, presents promising agronomic potential as a multinutrient, slow‐release fertilizer. This composition is particularly valuable for addressing potassium deficiencies in soil while minimizing the risk of traditional fertilizer burn (Rahman et al. 2014). Furthermore, the substantial removal of ammonium via pH‐induced volatilization reduces effluent toxicity, thereby enhancing the suitability of the treated wastewater for subsequent discharge or controlled agricultural reuse. Despite these advantages, the notable sodium content of the recovered hazenite presents an agronomic limitation. Unmanaged application of high‐sodium fertilizers carries risks of soil salinization and alkalinization, potentially degrading soil structure and impacting salt‐sensitive crops. Consequently, direct application may be constrained. Future research should evaluate postrecovery strategies, such as controlled washing or blending with low‐sodium amendments to manage the sodium adsorption ratio (SAR), alongside empirical greenhouse trials to assess actual soil impacts. However, translating these laboratory‐scale observations into industrial‐scale implementation necessitates addressing several methodological constraints. First, the initial 1:1 dilution was utilized to lower the high ionic strength of the livestock matrix and facilitate mechanical mixing. Thermodynamic evaluations based on the framework established in Section 2.5 indicate that although the absence of dilution increases the overall ionic strength and lowers activity coefficients, the proportionally higher precursor concentrations and the conserved molar surplus of alkali metals (K^+^ and Na^+^) continue to favor Hazenite formation. Under these conditions, the theoretical supersaturation index for Hazenite remains positive and higher than that of conventional struvite, suggesting that the observed phase shift is primarily driven by the intrinsic alkali‐rich stoichiometry of the matrix rather than dilution effects. Second, as the ammonia volatilization in this study was conducted in an open‐beaker configuration, industrial applications would require enclosed reactors equipped with acid scrubbers to ensure effective nitrogen recovery and to prevent atmospheric emissions. Furthermore, from an analytical perspective, the absence of a closed‐system mass balance or direct ammonia emission measurements in the current methodology restricts the volatilization mechanism to a well‐supported thermodynamic interpretation. Future empirical studies should incorporate acid‐trap off‐gas analysis to quantify the nitrogen partitioning and validate this theoretical stripping efficiency.

Finally, although chitosan effectively mitigated organic fouling and improved crystal purity, the 2000 mg/L dosage represents a potential challenge to economic scalability. To address this, future research should evaluate cost‐reduction strategies, such as dual‐coagulation systems combining chitosan with conventional metal salts or the integration of electrocoagulation. These technical pathways must be supported by a comprehensive techno‐economic assessment (TEA) and life cycle assessment (LCA) to determine the overall feasibility and environmental footprint of this integrated Hazenite recovery pathway.

## 4 Conclusion

This study demonstrates the feasibility of simultaneous macronutrient management from pretreated livestock wastewater, achieving solid‐phase crystal recovery for phosphorus, potassium, and magnesium, alongside secondary gaseous removal for nitrogen. Systematic evaluation established the optimal recovery conditions as pH 9.5, a Mg:N:P molar ratio of 1.2:1:1, and a temperature of 30°C. Under these parameters, the system achieved effective solid‐phase recovery efficiencies of 95% for orthophosphate and 91% for magnesium. Conversely, based on mass partitioning and solid‐phase characterization, the 97% removal of ammoniacal nitrogen is interpreted to be driven primarily by pH‐induced ammonia stripping rather than crystalline sequestration into the mineral lattice.

The integration of thermodynamic modeling and solid‐phase characterization suggests that this extensive volatilization depletes aqueous NH_4_^+^, shifting the precipitation pathway. Concurrently, strong competition from background alkali ions (K^+^ and Na^+^) facilitates the displacement of remaining ammonium from the crystal lattice, favoring alkali‐magnesium phosphate crystallization over standard struvite. Furthermore, calcium‐based precipitation remains kinetically inhibited by the high magnesium concentration and residual organic loads, restricting calcium integration to 2.76 wt% (1.47 At%).

Overall, the selective recovery of phosphorus into a Hazenite‐dominant phase provides a promising alternative for comprehensive nutrient upcycling, offering a multinutrient product with high potassium content. Future research should prioritize TEAs and LCAs to evaluate the industrial scalability and environmental footprint of this integrated valorization pathway, with a specific emphasis on evaluating the long‐term agronomic impacts and potential soil salinization risks associated with the high sodium content.

## Author Contributions

**Ahmet Demir:** supervision, project administration, writing – review and editing, conceptualization. **Zehra Çoban:** conceptualization, data curation, writing – original draft, formal analysis. **Ebru Koca Akkaya:** conceptualization, investigation, writing – review and editing, methodology. **Mehmet Sinan Bilgili:** conceptualization, methodology, writing – review and editing, investigation.

## Funding

This work has been supported by the Yildiz Teknik Üniversitesi (FBA‐2022‐5022).

## Conflicts of Interest

The authors declare no conflicts of interest.

## Supporting information


**Figure S1:** Optical microscopic images illustrating the macroscopic morphology of the synthesized precipitate.
**Table S1:** Comparison of removal and recovery yields and operating conditions with literature.
**Table S2:** Thermodynamic saturation indices (SI) of potential mineral phases.

## Data Availability

The datasets generated and/or analyzed during the current study are available from the corresponding author on reasonable request.
