# Supplementary material for: Nutrient Removal and Recovery: Hazenite Precipitation From Livestock Manure Wastewater
Source: Water Environ Res. 2026 Aug 13;98(8):e70529. doi: 10.1002/wer.70529 (PMC13470137; doi:10.1002/wer.70529)
Supplement: Supplementary file 1 [file WER-98-e70529-s001.doc]

**Supplementary Material**

**Nutrient Removal and Recovery: Hazenite Precipitation from Livestock Manure Wastewater**

**Figure S1.** Optical microscopic images illustrating the macroscopic morphology of the synthesized precipitate

**Table S1.** Comparison of removal and recovery yields and operating conditions with literature

| **Wastewater** | **Operating Conditions** | **Removal Efficiency (%)** | **Reference** |
| --- | --- | --- | --- |
| Dairy processing waste | pH 10  T= 10-20 °C  Mg:P=2.3:1 | >90 | [1] |
| Dairy industry wastewater | pH 9.6  Mg:N:P=1 | NH_4_=88  PO_4_=79 | [2] |
| Dairy industry wastewater | P:Mg=1:6 | P=80 | [3] |
| Source-diverted blackwater (feces and urine) | pH=9.0  Mg:P=1.3 | P=90 | [4] |
| Wastewater and natural water | T=25 ^o^C  pH=8,5  Mg:N:P = 1:1:1 | P removal in wastewater=70  P removal in natural water=54 | [5] |
| Swine wastewater | Seawater used as Mg source | P=80-90 | [6] |
| Simulated anaerobic digestate of swine wastewater | pH = 8.5  Mg:N:P = 1.2:5:1 | P removal almost 100 | [7] |
| Municipal wastewater | T= 20 °C  pH= 8-10  Mg:P=1.75  Mg:Ca=1.19:2:3 | P=85-95 | [8] |
| Poultry and multi-substrate agricultural waste digestates | pH=9  Mg:P=1.5 | NH_4_=95-99 | [9] |
| Cow digestate | pH=9,5  Mg:P=2 | P = 94  N = 66 | [10] |
| Non-digested wastewater from a manure composting plant | T=30 ^o^C  pH=9.5  Mg:NH_4_-N:PO_4_-P=1.2:1:1 | P= 95  N= 97  Mg= 91 | This Study |

**Table S2.** Thermodynamic saturation indices (SI) of potential mineral phases

| **Mineral Phase** | **Chemical Formula** | **Saturation Index (SI)** |
| --- | --- | --- |
| Hazenite | KNaMg_2_(PO_4_)_2_.14H_2_O | 2.10 |
| Struvite (MAP) | MgNH_4_PO_4_.6H_2_O | 1.38 |
| Trimagnesium phosphate | Mg_3_(PO_4_)_2_ | 2.83 |
| Brucite | Mg(OH)_2_ | 0.51 |
| Magnesium hydrogen phosphate | MgHPO_4_.3H_2_O | -0.28 |
| Periclase | MgO | -4.68 |
| Hydroxyapatite | Ca_5_(PO_4_)_3_(OH) | 21.92 |
| Amorphous calcium phosphate | Ca_3_(PO_4_)_2_ (am1) | 5.34 |

**References**

1.	McIntosh S, Hunt L, Thompson Brewster E, et al (2022) Struvite Production from Dairy Processing Waste. Sustain 14:. https://doi.org/10.3390/su142315807

2.	Uysal A, Kuru B (2015) The fertilizer effect of struvite recovered from dairy industry wastewater on the growth and nutrition of maize plant. Fresenius Environ Bull 24:3155–3162

3.	Purnomo CW, Kusumawardhani NP, Marpaung YN (2018) Phosphate recovery from dairy urine by struvite crystallization. AIP Conf Proc 2049:. https://doi.org/10.1063/1.5082409

4.	Sun H, Mohammed AN, Liu Y (2020) Phosphorus recovery from source-diverted blackwater through struvite precipitation. Sci Total Environ 743:140747. https://doi.org/10.1016/j.scitotenv.2020.140747

5.	Moulessehoul A, Harrache D, Gallart-Mateu D, et al (2024) Phosphorus removal and recovery from water and wastewater by the struvite crystallization. Desalin Water Treat 320:100902. https://doi.org/10.1016/j.dwt.2024.100902

6.	Gao D, Huang Y, Zhou S, et al (2024) Seawater boosts oxytetracycline (OTC) residues in struvite via hydroxyapatite (HAP) and dissolved organic matter (DOM) co-precipitation. J Hazard Mater 480:136355. https://doi.org/10.1016/j.jhazmat.2024.136355

7.	Song B, Wang R, Li W, et al (2024) Fate of micropollutants in struvite production from swine wastewater with sacrificial magnesium anode. J Hazard Mater 478:135505. https://doi.org/10.1016/j.jhazmat.2024.135505

8.	Santos AF, Abreu RL, Alvarenga P, et al (2024) An innovative two-step methodology for struvite recovery from wastewater with high purity and agronomic potential. J Water Process Eng 67:106206. https://doi.org/10.1016/j.jwpe.2024.106206

9.	Nagarajan A, Chen Y, Raghavan V, et al (2024) Sustainable nutrient recovery through struvite precipitation from poultry and multi-substrate agricultural waste digestates. Bioresour Technol Reports 27:101924. https://doi.org/10.1016/j.biteb.2024.101924

10.	Trotta S, Adani F, Fedele M, Salvatori M (2023) Nitrogen and phosphorus recovery from cow digestate by struvite precipitation: Process optimization to maximize phosphorus recovery. Results Eng 20:101478. https://doi.org/10.1016/j.rineng.2023.101478
